# Identification of two novel CT antigens and their capacity to elicit antibody response in hepatocellular carcinoma patients

**DOI:** 10.1038/sj.bjc.6601062

**Published:** 2003-07-15

**Authors:** X-Y Dong, Y-R Su, X-P Qian, X-A Yang, X-W Pang, H-Y Wu, W-F Chen

**Affiliations:** 1 Department of Immunology, School of Basic Medical Sciences, Peking University Health Science Center, 38 Xue Yuan Road, Beijing 100083, China

**Keywords:** CT antigen, hepatocellular carcinoma, antibody response

## Abstract

FATE and TPTE genes were originally reported to be specifically expressed in the adult testis. We searched for the databases of Unigene and serial analysis of gene expression (SAGE) implying that these two gene transcripts might also be expressed in tumours. Herein, we demonstrated that FATE and TPTE mRNA transcripts were expressed in different histological types of tumours and normal testis. Both are cancer-testis (CT) antigens and renamed as FATE/BJ-HCC-2 and TPTE/BJ-HCC-5, respectively. Comparison at nucleotide sequence, the FATE/BJ-HCC-2 cDNA, was identical to that of FATE, whereas the TPTE/BJ-HCC-5 was found to have two isoforms in both cancers and testis: one was identical in cDNA sequence to TPTE, encoding a protein of 551 amino acids, and the other variant lacked an exon of 54 bp, encoding a protein of 533 amino acids. The mRNA expression was analysed by RT–PCR and real-time PCR. FATE/BJ-HCC-2 mRNA was detected in 66% (41 out of 62) in hepatocellular carcinoma (HCC) samples and 21% (three out of 14) in colon cancer samples, whereas the TPTE/BJ-HCC-5 mRNA was detected in 39% (24 out of 62) and 36% (five out of 14) in HCC and non-small lung cancer samples, respectively. The recombinant proteins were prepared and the reactivity of allogenic sera to these two antigens was screened. The frequency of antibody response against FATE/BJ-HCC-2 and TPTE/BJ-HCC-5 proteins was 7.3% (three out of 41) and 25.0% (six out of 24), respectively, in HCC patients bearing respective gene transcripts. Therefore, FATE/BJ-HCC-2 and TPTE/BJ-HCC-5 are the novel CT antigens capable of eliciting antibody response in cancer patients.

Hepatocellular carcinoma (HCC) is one of the most common and severe diseases in the world (Okuda, 1992). Although there are many established therapeutic methods including surgery, chemotherapy, radiotherapy, ethanol injection therapy, and transarterial embolisation, the therapeutic results remain unsatisfactory due to late diagnosis.

The identification of tumour-associated antigens recognised by cellular and/or humoral effectors of the immune system has provided new perspectives for tumour therapy. A variety of tumour-associated antigens have been identified, including: cancer-testis (CT) antigens, which are expressed in different histological types of tumours, but not in normal tissues except testis, differentiation antigens, viral antigens, mutated antigens, and the products of overexpressed genes. Of all the antigens identified, the CT antigens are the most attractive targets for vaccination in tumour immunotherapy with the potential to eradicate residual tumour cells in multiple sites in the body. *In vitro* and *in vivo* experiments have demonstrated that some CT antigens, for example, NY-ESO-1, are capable of provoking potent T-cell-mediated immunity to directly kill tumour cells and/or release cytokines to interfere with the growth and propagation of tumour cells (Jager *et al*, 2001; Kirkin *et al*, 2002).

To date, a growing number of antigens have been cloned and identified by various approaches, such as SSX2 and NY-ESO-1 by SEREX (Sahin *et al*, 1995; Chen *et al*, 1997b; Güre *et al*, 1997), CT10 and SAGE (sarcoma-associated antigen) by RDA (Güre *et al*, 2000; Martelange *et al*, 2000), and CTp11 by differential mRNA expression (Zendman *et al*, 1999). Only a minority of the identified tumour antigens are immunogenic with the potential for immunotherapy. A crucial factor alleviating the efficacy of tumour immunotherapy is the immunoselection that occurs in the vaccination process and resulted in the progressive loss of tumour antigens together with the downregulation of HLA proteins (Giorgio *et al*, 2002). To improve the efficacy of tumour vaccines and prevent immune escape, further identification of novel CT antigens is warranted in order to develop applicable polyvalent tumour vaccines.

In this paper, we described two novel CT antigens identified by the analysis of databases and confirmed by experiments, termed FATE/BJ-HCC-2 and TPTE/BJ-HCC-5, respectively. Both of them are expressed, at a high percentage, in HCC and in some other types of tumours. The antibodies against these two antigens were detected in the sera of a minor proportion of HCC patients. It supports the fact that both CT antigens are immunogenic in cancer patients.

## MATERIALS AND METHODS

### Human tumour tissues and sera

All samples of human HCC, colon cancer, gastric cancer, and non-small cell lung cancer tissue and paired noncancerous tissue (5 cm away from tumour) were obtained during surgical resection from our 2nd Teaching Hospital, Peking University Health Science Center. Patients agreed to collection of tissue samples and sera with written consent and this was permitted by Hospital Ethic Review Committee. The resected tissue samples were immediately cut into small pieces, then, snap-frozen in liquid nitrogen until use. All tumour tissue and paired noncancerous tissue samples were pathologically confirmed. The patients from whom the resected HCC samples were obtained were HBV surface antigen positive, but HCV antigen negative.

### Unigene and SAGE databases analysis

Unigene is an experimental system for automatically partitioning GenBank sequences into a nonredundant set of gene-oriented clusters. Each Unigene cluster contains sequences that represent a Unigene gene, as well as the related information such as the tissue types in which the gene has been expressed and hundreds of thousands of expressed sequence tag (EST) sequences in which the tissue types are labelled (Schuler, 1997). Serial analysis of gene expression (SAGE) is an on-line technique that allows us to obtain a quantitative and comprehensive cellular gene expression profile (Velculescu *et al*, 1995). Consequently, Unigene and SAGE can be used to analyse the spatial and temporal expression pattern of a given gene for predicting the functional information of the gene.

### RT–PCR

The mRNA expression pattern was assayed by RT–PCR using panels of commercially available cDNA (Clontech, Palo Alto, CA, USA). One panel is comprised of 16 normal tissues: brain, heart, kidney, liver, lung, pancreas, placenta, skeletal muscle, colon, ovary, peripheral blood leucocyte, prostate, small intestine, spleen, testis, and thymus. RNA from HCC, paired adjacent noncancerous liver tissues and cancer tissues of lung, colon, and stomach was extracted, and reversely transcribed with Advantage reverse transcriptase (Clontech, Palo Alto, CA, USA). Gene-specific PCR primers were used to amplify cDNA fragments of FATE/BJ-HCC-2 and TPTE/BJ-HCC-5. RT–PCR was performed using the following parameters: FATE/BJ-HCC-2, 30 cycles, 94°C, 15 s; 68°C, 30 s; and 72°C, 30 s, followed by 6 min extension at 72°C; TPTE/BJ-HCC-5, 30 cycles, 94°C, 15 s; 65°C, 30 s; and 72°C, 1.5 min, followed by 6 min extension at 72°C. The sequence of paired primers for the amplification of FATE/BJ-HCC-2 and TPTE/BJ-HCC-5 was: FATE/BJ-HCC-2, forward: ctg ttc ctg gca ccc tgt gca tcc; reverse: gat gcc gcc atg ctg ttc acc c; TPTE/BJ-HCC-5, forward: cca caa tgg tcc acc cac aaa tg; reverse: aac atg tgg cag ggt tgg aaa gaa c.

### Quantification of gene expression

Quantification of FATE/BJ-HCC-2 and TPTE/BJ-HCC-5 expression was performed by monitoring the increase in fluorescence by the binding of SYBR green to double-stranded DNA during real-time PCR (Schmittgen *et al*, 2000). Their specific amplification primers were described above. All reactions were carried out in triplicate with positive and negative controls using the standard programme. Melt-curve analysis was performed to determine the temperature at which data were collected. For the data collection step in real-time PCR, after incubation at 50°C for 2 min, denaturing at 95°C for 10 min, the PCR parameters for FATE/BJ-HCC-2 were 40 cycles of 95°C, 15 s, 68°C, 1 min, and 86°C, 10 s; and the parameters for TPTE/BJ-HCC-5 were 40 cycles of 95°C, 15 s, 65°C, 2 min, and 88°C, 10 s. Gene expression level was measured by the quantification of cDNA relative to normal testis (Clontech, Palo Alto, CA, USA) serving as a calibrator sample and G3PDH gene was used as an endogenous control. To analyse the relative quantification for a target gene, the following data were required: the mean *C*_T_ value of the replicate wells running for each sample; the difference (Δ*C*_T_) between the mean *C*_T_ value of the samples for the target gene and of the samples for the endogenous controls; the difference (ΔΔ*C*_T_) between the Δ*C*_T_ values of the samples for each target and the mean *C*_T_ value of the calibrator for that target gene. The relative quantification value was expressed as 2^−ΔΔ*C*_T_^

### Northern blot analysis

Northern blots were performed with mRNA samples extracted from HCC and paired adjacent non-HCC liver tissues. RNA integrity was examined by electrophoresis in formalin/4-morpholine propane sulphonic acid gels. mRNA (2 *μ*g) per lane was first separated by electrophoresis in 1.2% agarose containing 3% formaldehyde and then blotted onto nylon membranes (Amersham Hybond-C, USA). The membranes were crosslinked under ultraviolet radiation. The DIG-labelled probes were the cDNA sequences of FATE/BJ-HCC-2 (506 bp) and TPTE/BJ-HCC-5 (1048 bp). After prehybridization, the membranes were hybridised to the specific DIG-labelled probes overnight at 42°C in hybridisation solution (50% formamide, 5XSSC, 0.1% *N*-lauroylsarcosine, 0.02% SDS, 2% blocking reagent, and 100 *μ*g ml^−1^ sheared salmon sperm DNA). Filters were then sequentially washed at room temperature for 30 min in 0.1% SDS, 2XSDS, and at 68°C for 30 min in 0.1% SDS and 0.1XSDS. After stringent washes, the corresponding mRNA in Northern blot was detected by chemiluminescence using CSPD ready-to-use reagent (Roche, Indianapolis, USA).

### Expression and purification of FATE/BJ-HCC-2 in *E. coli*

The fragment of ORF of FATE/BJ-HCC-2 was amplified with the template of the plasmid containing full-length cDNA and the primers of 5′-gcg gca tgc atg gca gga ggc cct ccc-3′ and 5′-gcg aag ctt tca ctg gtt cat cca cag cc-3′. The amplified products were digested with *Sph*I and *Hind*III, and the fragment was inserted into the expression plasmid pQE30, then, sequenced. The plasmid pQE30/FATE/BJ-HCC-2 was transformed into *E. coli* M15. After induction by 1 mM IPTG at 37°C for 6 h, the produced protein was purified by Ni^2+^ affinity chromatography, as the pQE30 expression plasmid encodes a 6 × His tag at the NH_2_-terminus.

### Expression and purification of TPTE/BJ-HCC-5 in insect cells

TPTE/BJ-HCC-5A and TPTE/BJ-HCC-5B cDNAs with *Bam*HI and *Hin*dIII restriction sites were cloned into the donor plasmid pFASTHTb. The recombinant plasmids pFASTHTb/TPTE/BJ-HCC-5A and pFASTHTb/TPTE/BJ-HCC-5B were introduced into competent cells DH10Bac. Recombinant bacmids were constructed by transposing a mini-Tn7 element from the donor plasmid pFASTHTb/TPTE/BJ-HCC-5A and pFASTHTb/TPTE/BJ-HCC-5B to the mini-attTn7 attachment site on the bacmids, where the Tn7 transposition function was provided *in trans* by a helper plasmid pMON7124. The bacmids were extracted, and transfected into sf9 insect cells to obtain recombinant baculvirus. Infection of sf9 insect cells was carried out in a serum-free medium of SFM at an m.o.i. of 10. Western blot (WB) analysis with anti-6 × His tag mAb was used to confirm the expression of recombinant TPTE/BJ-HCC-5A and TPTE/BJ-HCC-5B proteins fused with 6 × His tag. The purification of recombinant proteins was performed by Ni^2+^ affinity chromatography.

### Survey of the humoral immune response against FATE/BJ-HCC-2 and TPTE/BJ-HCC-5 antigens in the patients of hepatocellular carcinoma

The survey of the humoral immune response against FATE/BJ-HCC-2 and TPTE/BJ-HCC-5 antigens in HCC patients was performed by standard WB (Towbin *et al*, 1979) and enzyme-linked immunosorbent assay (ELISA).

### Western blot

Briefly, crude extracted FATE/BJ-HCC-2 and TPTE/BJ-HCC-5 proteins were separated on 10% SDS–polyacrylamide gel electrophoresis and transferred onto nitrocellulose membranes. After blocking with the TNT solution containing 5% dry milk, the membranes were sequentially incubated with HCC patients' sera diluted in 1 : 1000 for 1.5 h, then, incubated with goat anti-human IgG (H+L) alkaline phosphatase conjugate (Promega, Madison, WI, USA) for 1 h, and colour substrate NBT/BCIP (Promega) was added for 20-min colour development. The antibody-positive sera were further tested using purified recombinant proteins as antigens for verification. To test the specificity of Ab response against FATE/BJ-HCC-2 and TPTE/BJ-HCC-5 proteins, the irrelevant proteins of BJ-9 expressed in *E. coli* and HCA587 expressed in insect cells were applied in WB assay using the HCC patient's sera, in which the antibody was detected against FATE/BJ-HCC-2 or TPTE/BJ-HCC-5, but not to BJ-9 or HCA587 (Wang *et al*, 2002). Mock-transfected *E. coli* and insect cell lysates were used in WB as negative controls.

### ELISA

Recombinant proteins of FATE/BJ-HCC-2 and TPTE/BJ-HCC-5 at a concentration of 1 *μ*g ml^−1^ in coating buffer (15 mM Na_2_CO_3_, 30 mM NaHCO_3_, pH 9.6 with 0.02% NaN_3_) were absorbed onto 96-well Munc-Immuon plates (Nunc. Polysorp, Denmark) at 100 *μ*l per well overnight at 4°C, respectively. Plates were washed with 0.05% Tween-20/PBS and blocked for 1 h at room temperature (RT) with 100 *μ*l per well of 2% BSA–PBS. After washing, 100 *μ*l per well of a serial dilution of serum in 2% BSA–PBS was added and incubated for 2 h at 37°C in a water-saturated incubator. Plates were washed and 100 *μ*l per well diluted secondary Ab in 2% BSA–PBS was added (Goat anti-human IgG-AP: Southern Biotechnology, Birmingham, AL, USA) and incubated for 1 h at 37°C. Plates were washed, incubated with 100 *μ*l per well of substrate solution (*p*-nitrophenyl phosphate, Sigma-Aldrich, Inc., USA) for 20–30 min at RT, then the reaction was stopped by adding 3 N NaOH 50 *μ*l per well and immediately read at 410 nm (ELISA Reader, BIO-RAD, M550, USA). For the serological survey of human sera, the serum of melanoma patient NW29 was used as a positive control for NY-ESO-1 and also as a negative control for MAGE-1 (provided by Ludwig institute for Cancer Research, NY, USA). All the sera were tested over a range of serial four-fold dilution from 1 : 100 to 1 : 102 400. A positive reaction is defined as an OD value of a 1 : 400 diluted serum that exceeds the mean OD value of negative control (coated with irrelevant protein) by 3 s.d.

## RESULTS

### Databases analysis

In our analysis, three criteria were used to limit the candidate genes. First, the candidate genes are definitely testis-specific, but they have not been reported to be expressed in tumours. Second, there must have been ESTs expressed in tumours for the candidate genes in the Unigene database. Third, if there are SAGE tags for the sequences of the candidate genes, only the tags absolutely corresponding to the candidate genes are included, because there are lots of SAGE tags that are not only homologous to the candidate genes but also represent other genes.

When these criteria were applied, two genes were found to be the most promising candidates for CT antigen-encoding genes: fetal and adult testis expressed transcript (FATE) and transmembrane phosphatase with tensin homology (TPTE). Both of them are known as testis-specific genes. In the database of Unigene, there were 33 and 47 ESTs representing FATE and TPTE, respectively. Although most of these ESTs were identified from the libraries of testis, two ESTs for FATE were derived from adrenal gland carcinoma and adrenal cortico-adenoma for Cushing's syndrome, and four ESTs for TPTE derived from adrenal gland carcinoma, uterus tumour and colon cancers. In the SAGE database, two tags representing TPTE were identified from the libraries of brain glioblastoma multiform cell line and ovary carcinoma, although no tag for FATE was found. We proposed the FATE and TPTE as the potential CT antigen encoding genes and renamed them as FATE/BJ-HCC-2 and TPTE/BJ-HCC-5.

### Expression profile of FATE/BJ-HCC-2 and TPTE/BJ-HCC-5

The mRNA expression profile of FATE/BJ-HCC-2 and TPTE/BJ-HCC-5 was determined by RT–PCR. A series of tissues and cell lines were examined, including normal tissues, tumour cell lines, HCC and corresponding adjacent noncancerous tissues, gastric cancer, non-small cell lung cancer, and colon cancer tissues.

### Expression in normal tissues

**Figure 1:**
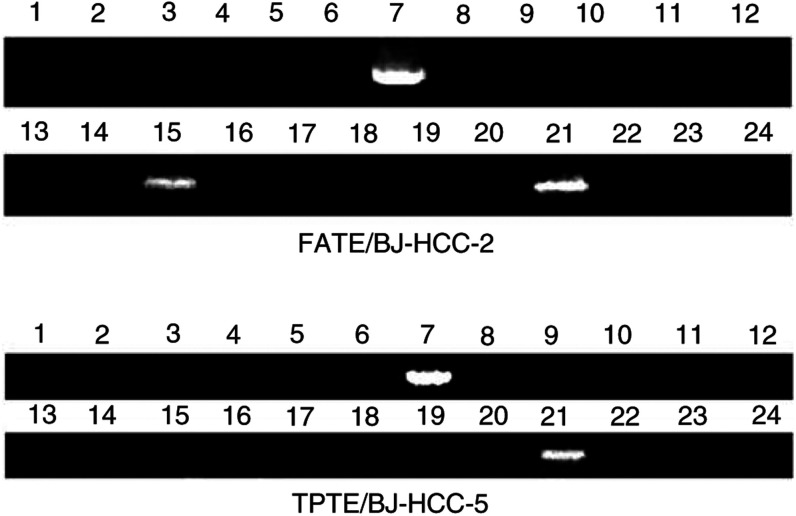
Expression of FATE/BJ-HCC-2 and TPTE/BJ-HCC-5 mRNA in 16 normal tissues and eight cancer cell lines. Gel electrophoresis of RT–PCR products shows FATE/BJ-HCC-2 and TPTE/BJ-HCC-5 transcripts expressed in testis, normal pancreas, and in some cancer cell lines. 1–16, normal tissues: (1) brain, (2) heart, (3) kidney, (4) liver, (5) lung, (6) placenta, (7) testis, (8) skeletal muscle, (9) colon, (10) ovary, (11) leucocytes, (12) prostate, (13) small intestine, (14) spleen, (15) pancreas, (16) thymus. (17–24), cancer cell lines: (17) breast carcinoma (GI-101), (18) lung carcinoma (LX-1), (19) colon adenocarcinoma (CX-1), (20) lung carcinoma (GI-117), (21) prostatic adenocarcinoma (PC3), (22) colon adenocarcinoma (GI-112), (23), ovarian carcinoma (GI-102), (24), pancreatic adenocarcinoma (GI-103).

For the detection of mRNA expression of FATE/BJ-HCC-2, there were 16 different normal tissues of spleen, prostate, testis, ovary, small intestine, colon, peripheral blood, heart, lung, liver, adrenal gland, whole brain, kidney, pancreas, placenta, and skeletal muscle being tested by RT–PCR (Olesen *et al*, 2001), FATE/BJ-HCC-2 was strongly expressed in testis, and weakly detected in pancreatic tissue, but undetectable in other tissues. Quantification of gene expression revealed that the expression level of FATE/BJ-HCC-2 mRNA in the testis was five-fold higher than that in the pancreas. For the detection of mRNA expression of TPTE/BJ-HCC-5, there were 18 different normal tissues of spleen, thymus, testis, small intestine, heart, lung, liver, brain, kidney, skin, stomach and skeletal muscle, prostate, ovary, colon, peripheral blood, pancreas, and placenta being tested by RT–PCR. Like the previous report (Wu *et al*, 2001), the TPTE/BJ-HCC-5 mRNA was detected in the testis only (Figure 1

).

### Expression in cancer cell lines

A panel of eight cancer cell lines (breast, G1-101; lung, LX-1 and G1-117; colon, CX-1 and G1-112; prostate, PC3; ovary, G1-102; and pancreas, G1-103) were tested. Both FATE/BJ-HCC-2 and TPTE/BJ-HCC-5 were only expressed in prostate adenocarcinoma cell line PC3 (Figure 1).

### Expression in HCC tissues, adjacent noncancerous liver tissues, cirrhosis, and other cancer tissues

**Figure 2:**
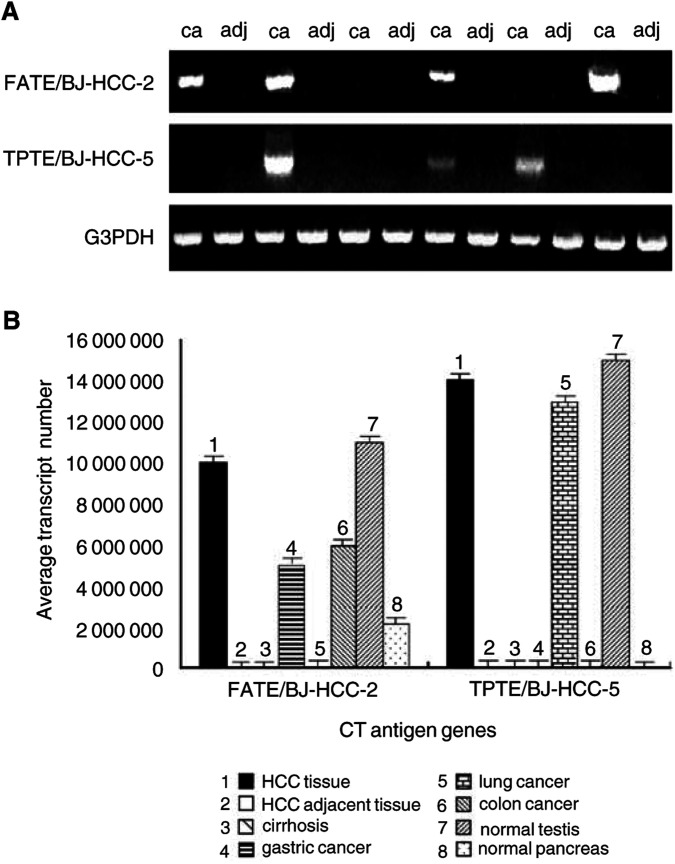
Expression of FATE/BJ-HCC-2 and TPTE/BJ-HCC-5 mRNA transcripts in six paired samples of HCC and adjacent noncancerous liver tissues. (**A**) Gel electrophoresis of RT–PCR products shows FATE/BJ-HCC-2 and TPTE/BJ-HCC-5 mRNA transcripts in the paired HCC and adjacent noncancerous liver tissue samples. RT–PCR for G3PDH was used to monitor the quality of the mRNA samples. (**B**) Real-Time PCR quantification of the expression of FATE/BJ-HCC-2 and TPTE/BJ-HCC-5 mRNA transcripts in HCC tissues, paired adjacent noncancerous liver tissues, cirrhotic liver tissues, gastric cancer, non-small cell lung cancer and colon cancer tissue samples, normal testis, and pancreas.

FATE/BJ-HCC-2 and TPTE/BJ-HCC-5 mRNA transcripts were analysed for their expression in the samples of HCC tissues and other cancer tissues. The FATE/BJ-HCC-2 mRNA transcript was substantially expressed in 41 out of 62 HCC samples (66%), weakly expressed in 12 out of 62 corresponding adjacent noncancerous liver tissues, but not expressed in 10 liver samples of cirrhosis. The average quantification of FATE/BJ-HCC-2 mRNA transcript was 120-fold higher in HCC tissues than in corresponding adjacent noncancerous liver tissues. TPTE/BJ-HCC-5 mRNA transcript was specifically expressed in 24 out of 62 HCC samples (39%), with no detection in corresponding adjacent noncancerous liver tissues or in cirrhotic liver samples. The average quantification of TPTE/BJ-HCC-5 mRNA expression in tumour samples was approximately comparable to that in the testis (Figure 2

). To assess whether FATE/BJ-HCC-2 and TPTE/BJ-HCC-5 mRNA transcripts were expressed in different histological types of tumours, their mRNA expression in gastric cancer, colon cancer, and non-small cell lung cancer tissues was tested. FATE/BJ-HCC-2 was expressed in 7% (one out of 14) gastric cancer and 21% (three out of 14) colon cancer samples, but not detected in 14 non-small cell lung cancer samples. TPTE/BJ-HCC-5 was expressed in 36% (five out of 14) non-small cell lung cancer samples, but not detected in 14 gastric cancer and 14 colon cancer samples. Thus, both FATE/BJ-HCC-2 and TPTE/BJ-HCC-5 were expressed in normal testis and certain types of cancers.

### Isolation of full-length cDNA of BJ-HCC-2 and BJ-HCC-5 from HCC tissues

**Figure 3:**
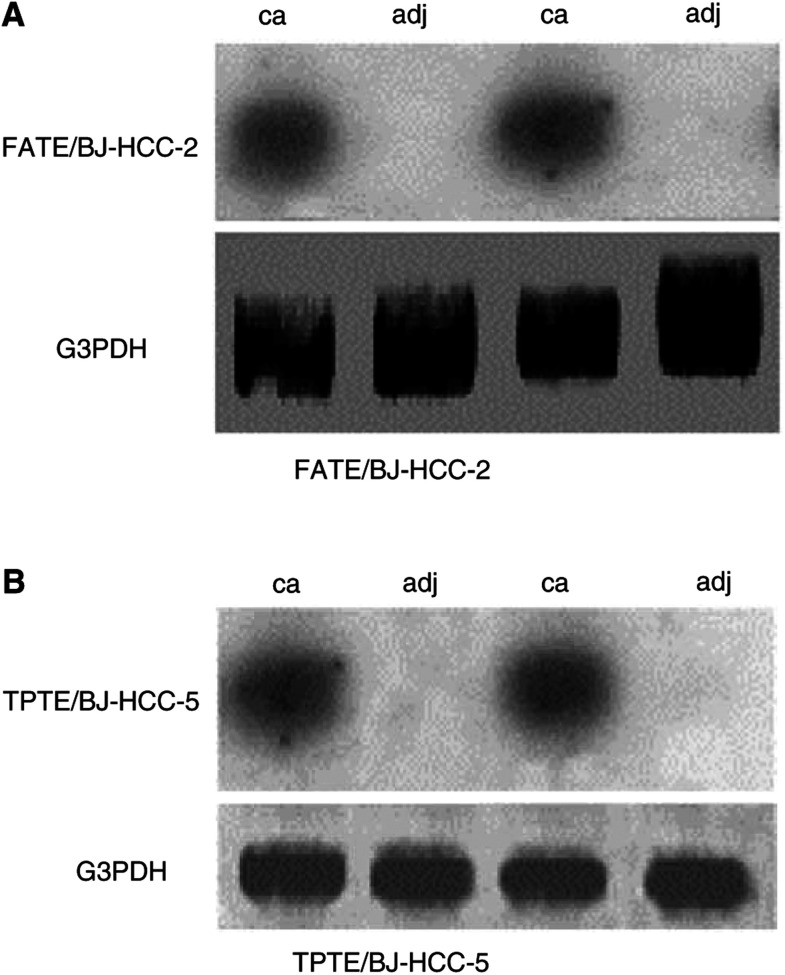
Northern blot hybridisation of FATE/BJ-HCC-2 and TPTE/BJ-HCC-5 in two pairs of HCC and adjacent noncancerous liver tissues samples. In the Northern blot analysis, FATE/BJ-HCC-2 and TPTE/BJ-HCC-5 mRNAs were only expressed in HCC tissues. The sizes of FATE/BJ-HCC-2 and TPTE/BJ-HCC-5 mRNA transcripts were 1.1 and 2.5 kb, respectively.

**Figure 4:**
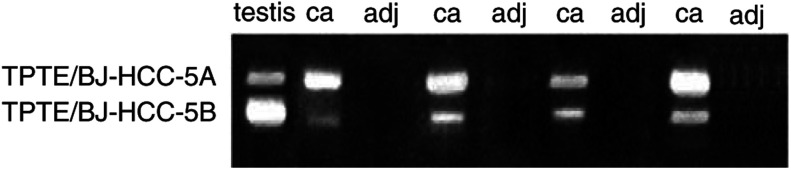
Two isoforms of TPTE/BJ-HCC-5. A pair of specific primers spanning the exon was designed and RT–PCR was performed using TPTE/BJ-HCC-5 cDNA as the template. Gel electrophoresis of RT–PCR products shows two TPTE/BJ-HCC-5 mRNA transcripts.

To further examine the RT–PCR results for FATE/BJ-HCC-2 and TPTE/BJ-HCC-5, mRNA was extracted from HCC and corresponding adjacent noncancerous liver tissues, then, analysed by Northern blot (Figure 3

). The hybridisation signal of FATE/BJ-HCC-2 was shown as the size of 1.1 kb, whereas the TPTE/BJ-HCC-5 was shown as the size of 2.5 kb in Northern blot, respectively, consistent with the transcripts expressed in the testis. Gene-specific primers (5′-aat tta gct gcg cac agg gag gtg-3′ and 5′-tga gcg tgt tgt ctg tgc aga gcg-3′ for FATE/BJ-HCC-2; 5′-cca caa tgg tcc acc cac aaa tg-3′ and 5′-aac atg tgg cag ggt tgg aaa gaa c-3′ for TPTE/BJ-HCC-5) were designed to isolate the full-length cDNAs of FATE/BJ-HCC-2 and TPTE/BJ-HCC-5 from HCC. Sequence analysis of the PCR product revealed that FATE/BJ-HCC-2 was identical to FATE in cDNA sequence. However, TPTE/BJ-HCC-5 had two different sequences: one was identical to TPTE (1769 bp) and the other was 54 bp shorter than TPTE. DNA alignment indicated that the shorter TPTE/BJ-HCC-5 lacked an exon of 54 bp than TPTE. A pair of primers (5′-cca caa tgg tcc acc cac aaa tg-3′ and 5′-agg tcg gca agg atg aga gtg aca tc-3′) was then designed to specifically amplify the cDNA fragments spanning the exon in all samples by RT–PCR, and the TPTE/BJ-HCC-5 mRNA transcripts were clearly shown as two bands simultaneously with the sizes of 390 and 336 bp, respectively (Figure 4

). The two PCR products were sequenced and the results were those as predicted. Therefore, TPTE/BJ-HCC-5 mRNA was expressed as two isoforms: one was identical to TPTE, which encoded a protein of 551 amino acids, called TPTE/BJ-HCC-5A, and the other was the alternative splice variant of TPTE, which lacked the sequence of an exon (54 bp) and encoded a protein of 533 amino acids, lacking 18 amino acids at the position of 41–58 in the NH_2_-terminus of TPTE, called TPTE/BJ-HCC-5B (GenBank Accession Number AF495908). Both TPTE isoforms were expressed in normal testis and cancers, with the TPTE/BJ-HCC-5B being dominant in testis and the TPTE/BJ-HCC-5A dominant in cancers. The deduced protein of FATE/BJ-HCC-2 was 183 amino acids in length with an isoelectric point of 9.6. FATE/BJ-HCC-2 was predicted to be a soluble protein, but had no signal peptide or motif with known function. There were three *N*-myristoylation sites, one *N*-glycosylation site, one glycosaminoglycan attachment site, and several kinase phosphorylation sites. The TPTE/BJ-HCC-5 possessed four potential transmembrane domains shown in hydropathy analysis and had an isoelectric point of 8.5. There were two potential *N*-myristoylation sites, two *N*-glycosylation sites, one tyrosine-specific protein phosphatases active site, and several kinase phosphorylation sites in TPTE/BJ-HCC-5 proteins. Both FATE/BJ-HCC-2 and TPTE/BJ-HCC-5 may undergo post-translational modifications. By chromosomal mapping, FATE/BJ-HCC-2 gene was located to chromosome Xq28, where a lot of important CT antigen encoding genes are located, such as MAGE family and NY-ESO-1 (De Plaen *et al*, 1994; Chen *et al*, 1997a). By contrast, the TPTE/BJ-HCC-5 may be located in the human chromosomes 13, 15, 21, 22, and Y, indicating that this gene might be present in different forms, some of which appeared to be pseudogenes (Chen *et al*, 1999).

### Expression and purification of FATE/BJ-HCC-2 and TPTE/BJ-HCC-5 proteins

In order to analyse the antibody response to FATE/BJ-HCC-2 and TPTE/BJ-HCC-5 proteins in HCC patients, we expressed the respective recombinant proteins. The FATE/BJ-HCC-2 protein was expressed in *E.coli* and accounted for 25% of the total protein. TPTE/BJ-HCC-5 proteins was failed to be expressed in *E. coli*, but expressed in insect cells using the Bac-to-Bac expression system. The expression level was 0.2 mg in the cultures of 10^7^ insect cells. Both recombinant proteins contained 6 × His tag to facilitate protein identification.

### A survey of sero-reactivity against FATE/BJ-HCC-2 and TPTE/BJ-HCC-5 proteins in HCC patients

**Figure 5:**
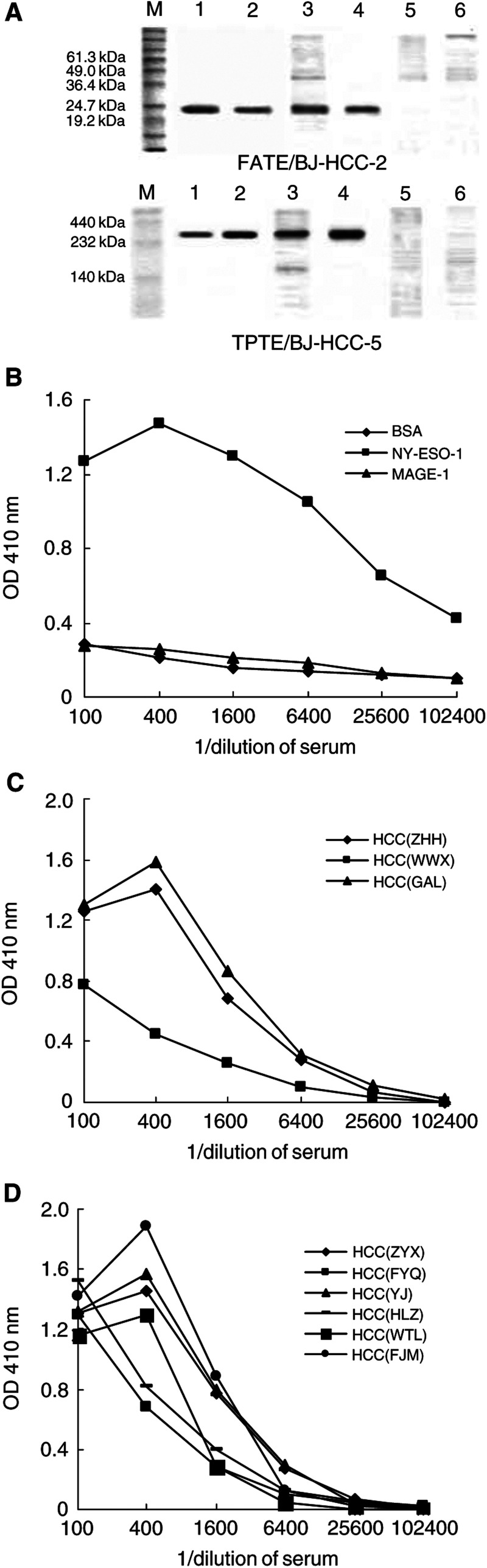
Antibody response against FATE/BJ-HCC-2 and TPTE/BJ-HCC-5 recombinant proteins in the sera of HCC patients. (**A**) Western blot analysis of the positive sera against FATE/BJ-HCC-2 and TPTE/BJ-HCC-5. The proteins of BJ-9 produced in *E. coli* and HCA587 produced in insect cells were applied as irrelevant antigens in the WB assays. Lanes 1–6 correspond to lysates of *E. coli* (or insect cells) containing recombinant proteins with 6 × His tag mAb, purified recombinant proteins with 6 × His tag mAb, lysates of *E. coli* (or insect cells) containing recombinant proteins with the positive sera, purified recombinant proteins with the positive sera, lysates of *E. coli* (or insect cells) containing irrelevant protein controls with the positive sera, and the negative controls of *E. coli* (or insect cells) with the positive sera. Lanes 1, 3, 5, and 6 contained 100 *μ*g lysates of *E. coli* or insect cells, whereas lanes 2 and 4 contained 5 *μ*g purified recombinant proteins. Lane M corresponds to protein marker. (**B**) The standardisation of ELISA. A standard serum obtained from a melanoma patient NW29 was antibody positive against NY-ESO-1, with no detectable Ab against MAGE-1. The titre of Ab against NY-ESO-1 was 1 : 6400 measured by indirect ELISA. (**C**) Antibody titre against FATE/BJ-HCC-2 measured by indirect ELISA. The three positive sera with the Ab against FATE/BJ-HCC-2 assessed by WB were measured by ELISA. The Ab titre were 1 : 6400, 1 : 1600, and 1 : 6400, respectively. (**D**) Antibody titre against FATE/BJ-HCC-5 measured by indirect ELISA. The six positive sera with the Ab against FATE/BJ-HCC-5 assessed by WB were measured by ELISA. The Ab titre was in the range around 1 : 1600–1 : 3200.

Sero-reactivity was firstly screened using WB analysis. In the 62 sera collected from HCC patients, there were three sera reactive to FATE/BJ-HCC-2 protein and six sera to TPTE/BJ-HCC-5 proteins (Figure 5A

). As the specificity controls, BJ-9 proteins expressed in *E. coli* and HCA587 expressed in insect cells were applied as irrelevant proteins in WB using the sera Ab positive to FATE/BJ-HCC-2 and FATE/BJ-HCC-5, respectively. The sero-reactivity was negative to BJ-9 and HCA587. In the 18 sera collected from normal individuals, none was reactive to FATE/BJ-HCC-2 or FATE/BJ-HCC-5 protein. The WB analysis was repeated twice and the same results were obtained. To further confirm the Ab response and semiquantitate the Ab titre, indirect ELISA was applied. The ELISA was optimised using the serum of melanoma patient NW29 as the standard serum, in which the Ab against NY-ESO-1 was positive, but with no detectable Ab against MAGE-1 (Figure 5B). In the three positive sera with the Ab against FATE/BJ-HCC-2, the Ab titre was 1 : 6400, 1 : 1600, and 1 : 6400, respectively (Figure 5C). In the six positive sera with the Ab against FATE/BJ-HCC-5, the Ab titre was in the range around 1 : 1600–1 : 3200 (Figure 5D). The Ab was only detected in the HCC patients whose resected tumours expressed the FATE/BJ-HCC-2 or TPTE/BJ-HCC-5 mRNA, not in the HCC patients bearing FATE/BJ-HCC-2 or TPTE/BJ-HCC-5 mRNA negative tumours. Therefore, the actual frequency of antibody response against FATE/BJ-HCC-2 and TPTE/BJ-HCC-5 proteins was 7.3% (three out of 41) and 25.0% (six out of 24), respectively, in HCC patients bearing respective gene transcripts.

## DISCUSSION

In this study, we targeted known testis-specific genes to investigate the possibility that some of these would be the CT antigen-encoding genes by database analysis. The selected candidates were assayed for their mRNA expression profile in normal tissues, tumour cell lines, HCC tissues and corresponding adjacent noncancerous liver tissues, and the tissues of gastric cancer, non-small cell lung cancer, and colon cancer. Two known testis-specific genes, FATE and TPTE, were identified to be restrictedly expressed in different cancers and normal testis. The FATE was also weakly expressed in normal pancreatic tissue. The TPTE gene was originally reported to encode a protein of 551 amino acids (Chen *et al*, 1999). We identified an alternative splice variant of TPTE cDNA, which was otherwise identical to TPTE except lacking an exon (54 bp), encoding a protein of 533 amino acids. Its sequence at the exon–intron junction is consistent with the AG-GT rule. For distinction, we called the TPTE as TPTE/BJ-HCC-5A and the variant as the TPTE/BJ-HCC-5B. These two isoforms of TPTE cDNA might be caused by different splicing, and the splicing machinery might be regulated by different factors that resulted in the TPTE/BJ-HCC-5B dominantly expressed in testis and the TPTE/BJ-HCC-5A dominantly expressed in cancer tissues. Serological survey of 62 sera collected from HCC patients revealed that 4.8% (three out of 62) and 9.6% (six out of 62) had antibody response to FATE/BJ-HCC-2 protein and TPTE/BJ-HCC-5 proteins, respectively. Regarding to the fact that Ab response was only detected in the patients bearing FATE/BJ-HCC-2 or TPTE/BJ-HCC-5 mRNA^+^HCC, the correct frequency of Ab response against FATE/BJ-HCC-2 and TPTE/BJ-HCC-5 proteins was 7.3% (three out of 41) and 25.0% (six out of 24), respectively. Thus, the FATE/BJ-HCC-2 and TPTE/BJ-HCC-5 proteins are immunogenic and can be classified as the novel CT antigens.

Apart from a few CT antigens, such as SCP-1, a meiosis protein (Heyting, 1996; Tureci *et al*, 1998), SSX, the transcriptional repressors (Brett *et al*, 1997), OY-TES-1, a proacrosin binding protein (Baba *et al*, 1994), HOM-TES-85, a novel leucine zipper protein, the biological function of the majority of CT antigens is unknown. FATE/BJ-HCC-2 was found to be coexpressed with SRY, which functions as a genetic switch that directs the development of the indifferent gonad from a female to a male pathway, in the 7-week-old fetal testis and might play a role in early testicular differentiation (Olesen *et al*, 2001). The expression of FATE/BJ-HCC-2 is regulated by steroidogenic factor (SF-1) and Wilms' tumour gene 1 (WT1) and involved in gamesomeness. As SF-1 and WT1 are involved in the development of tumour (Bamberger *et al*, 1996; Menke *et al*, 1998; Lopez *et al*, 1999; Sugiyama, 2001; Aylwin *et al*, 2001; Oka *et al*, 2002), the FATE/BJ-HCC-2 may also be involved in tumourigenesis. The TPTE/BJ-HCC-5 contributes to the terminal stages of spermatocyte differentiation (Wu *et al*, 2001). The TPTE/BJ-HCC-5A and TPTE/BJ-HCC-5B share significant homology with tumour suppressor protein PTEN, which is a phosphatidylinositol phospholipid phosphatase (Maehama *et al*, 1998). TPTE/BJ-HCC-5A and TPTE/BJ-HCC-5B are also phospholipid phosphatases and are localised on the membrane of the Golgi complex (Wu *et al*, 2001). Although lacking 18 amino acids in the NH_2_-terminus, the altered TPTE/BJ-HCC-5B has all the important functional domains of TPTE/BJ-HCC-5A. In view of the fact that many tumour-derived missense mutations were observed in PTEN resulting in cell cycle progression and inhibition of apoptosis, the TPTE/BJ-HCC-5A and TPTE/BJ-HCC-5B may be involved in the survival of tumour cells. TPTE/BJ-HCC-5A and TPTE/BJ-HCC-5B contribute to the terminal stages of spermatocyte differentiation (Wu *et al*, 2001). With respect to the fact that CT antigen proteins are expressed in both testis and cancers, they may have functional similarities in the spermatogenesis and tumourigenesis. The functional clues of FATE/BJ-HCC-2 and TPTE/BJ-HCC-5 make them the molecular models for analysing the shared mechanism between gametogenesis and tumourigenesis.

In conclusion, both FATE/BJ-HCC-2 and TPTE/BJ-HCC-5 are novel CT antigens. Further analysis needs to be carried out to investigate their capacity in the elicitation of specific cell-mediated immunity to determine if they are potential candidates for tumour immunotherapy.
